# snRNAs from Radical Prostatectomy Specimens Have the Potential to Serve as Prognostic Factors for Clinical Recurrence after Biochemical Recurrence in Patients with High-Risk Prostate Cancer

**DOI:** 10.3390/cancers16091757

**Published:** 2024-05-01

**Authors:** Hikaru Mikami, Syunya Noguchi, Jun Akatsuka, Hiroya Hasegawa, Kotaro Obayashi, Hayato Takeda, Yuki Endo, Yuka Toyama, Hiroyuki Takei, Go Kimura, Yukihiro Kondo, Toshihiro Takizawa

**Affiliations:** 1 Department of Urology, Nippon Medical School Hospital, Tokyo 113-8603, Japan; h-mikami86@nms.ac.jp (H.M.); s00-001@nms.ac.jp (J.A.); h-hasegawa@nms.ac.jp (H.H.); kotaro-o@nms.ac.jp (K.O.); s8053@nms.ac.jp (H.T.); y-endo1@nms.ac.jp (Y.E.); s4036@nms.ac.jp (Y.T.); gokimura@nms.ac.jp (G.K.); kondoy@nms.ac.jp (Y.K.); 2 Department of Molecular Medicine and Anatomy, Nippon Medical School, Tokyo 113-8602, Japan; n-syunya@nms.ac.jp; 3 Department of Breast Surgical Oncology, Nippon Medical School, Tokyo 113-8602, Japan; takei-hiroyuki@nms.ac.jp

**Keywords:** prostate cancer, radical prostatectomy, biochemical recurrence, clinical recurrence, prognostic factor, snRNA, formalin-fixed paraffin-embedded sample, RNA sequencing

## Abstract

**Simple Summary:**

In patients with high-risk prostate cancer (HRPC) after radical prostatectomy, biochemical recurrence increases the risk of distant metastasis. Therefore, complementary prognostic biomarkers are required to identify the subpopulation of patients with HRPC who develop clinical recurrence after biochemical recurrence. This study was performed to identify prognostic factors for clinical recurrence in patients with HRPC who experience biochemical recurrence by conducting an analysis of the expression levels of snRNAs in formalin-fixed paraffin-embedded (FFPE) radical prostatectomy samples. The FFPE sample-derived snRNA RNU1-1/RNU1-2 could serve as an independent prognostic factor of clinical recurrence-free survival after biochemical recurrence of HRPC cases where known prognostic factors (e.g., Gleason score) cannot distinguish between patients with clinical and non-clinical recurrence. Thus, snRNAs associated with prostate cancer may assist the early detection of clinical recurrence in patients with HRPC, allowing for more tailored and restorative treatments.

**Abstract:**

In patients with high-risk prostate cancer (HRPC) after radical prostatectomy (RP), biochemical recurrence (BCR) increases the risk of distant metastasis. Accordingly, additional prognostic biomarkers are required to identify the subpopulation of patients with HRPC who develop clinical recurrence (CR) after BCR. The objective of this study was to identify biomarkers in formalin-fixed paraffin-embedded (FFPE) RP samples that are prognostic for CR in patients with HRPC who experience BCR after RP (post-RP BCR). First, we performed a preliminary RNA sequencing analysis to comprehensively profile RNA expression in FFPE RP samples obtained from patients with HRPC who developed CR after post-RP BCR and found that many snRNAs were very abundant in preserved FFPE samples. Subsequently, we used quantitative polymerase chain reaction (qPCR) to compare the expression levels of highly abundant snRNAs in FFPE RP samples from patients with HRPC with and without CR after post-RP BCR (21 CR patients and 46 non-CR patients who had more than 5 years of follow-up after BCR). The qPCR analysis revealed that the expression levels of snRNA RNU1-1/1-2 and *RNU4-1* were significantly higher in patients with CR than in patients without CR. These snRNAs were significantly correlated with clinical recurrence-free survival (RFS) in patients with HRPC who experienced post-RP BCR. Furthermore, snRNA RNU1-1/1-2 could serve as an independent prognostic factor for clinical RFS in post-RP BCR of HRPC cases where known prognostic factors (e.g., Gleason score) cannot distinguish between CR and non-CR patients. Our findings provide new insights into the involvement of snRNAs in prostate cancer progression.

## 1. Introduction

Prostate cancer (PC), the second most common male cancer, is an important global health issue; worldwide incidence and mortality rates have been increasing over the past couple of decades [1,2,3]. Radical prostatectomy (RP), a definitive therapy for PC, is recommended for certain patients with high-risk PC (HRPC; prostate-specific antigen (PSA) ≥ 20 ng/mL, Gleason score (GS) ≥ 8, or clinical stage ≥ cT3a) [4,5]. Although RP is highly effective, such patients have a higher risk of recurrence and progression after RP compared with patients exhibiting low- and intermediate-risk PC [6,7,8,9,10,11,12]. After RP, a detectable serum PSA level of at least 0.2 ng/mL is considered indicative of biochemical recurrence (BCR); the presence of metastases on imaging after BCR is diagnostic of clinical recurrence (CR) [10,13,14]. Among all patients with HRPC after RP, 46% experience BCR (designated as post-RP BCR in patients with HRPC); moreover, the 10-year PC-specific mortality rate among patients with HRPC who experience post-RP BCR can reach 9% [15]. Both statistical and clinical indicators show that certain patients with HRPC who experience post-RP BCR have a high risk of CR [9,16,17]. Thus, patients with HRPC who experience post-RP BCR require dedicated management and surveillance according to current risk stratification methods: pathological-grade group, PSA doubling time, and molecular imaging data [15,18,19,20]. An accurate prediction of such a patient subgroup that will develop metastatic progression (i.e., CR) or die of PC remains challenging. Additional prognostic biomarkers are required to reveal the subpopulation of patients with HRPC who may develop CR after post-RP BCR during extended follow-up and thus need second-line treatments (i.e., radiation/hormone therapy).

Formalin-fixed paraffin-embedded (FFPE) PC tissues obtained during RP constitute a critical resource in terms of the pathological diagnosis of PC. A pathological assessment of FFPE RP samples is important for guiding treatment decisions and predicting patient outcomes (e.g., PC-specific mortality) [21,22]. A more accurate evaluation of GS using FFPE RP samples is important for PC risk management [23]. FFPE RP samples serve as a valuable resource for molecular characterization of PC and biomarker discovery through in situ detection and/or extraction of FFPE biomolecules (e.g., nucleic acids, proteins, and metabolites), facilitating the comprehension of PC progression and aggressiveness [24,25,26,27].

Small nuclear RNAs (snRNAs) are small non-coding RNAs (~150 nucleotides in length) found in the nucleus. snRNAs serve as the RNA components of the spliceosome that recognizes 5′ and 3′ intron/exon junctions during intron splicing; they play essential roles in the processing of pre-mRNAs [28,29,30]. snRNAs have recently received attention as potential biomarkers of certain types of cancer [31,32,33]. However, very little is known about the diagnostic and prognostic utilities of snRNAs in PC.

The objective of this study was to identify new prognostic factors for CR in patients with HRPC who experience post-RP BCR, using FFPE RP samples. First, we performed a preliminary RNA sequencing analysis to comprehensively profile RNA expression in FFPE RP samples from patients with HRPC who developed CR after post-RP BCR. We found that many snRNAs were very abundant in preserved FFPE samples. Next, we used quantitative polymerase chain reaction (qPCR) to compare the expression levels of highly abundant snRNAs in FFPE RP samples between HRPC groups with and without CR after post-RP BCR. We evaluated the potential utilities of snRNAs as novel prognostic indicators of metastatic potential in patients with HRPC who experienced post-RP BCR.

## 2. Materials and Methods

### 2.1. Patient Selection and Study Design

**Figure 1:**
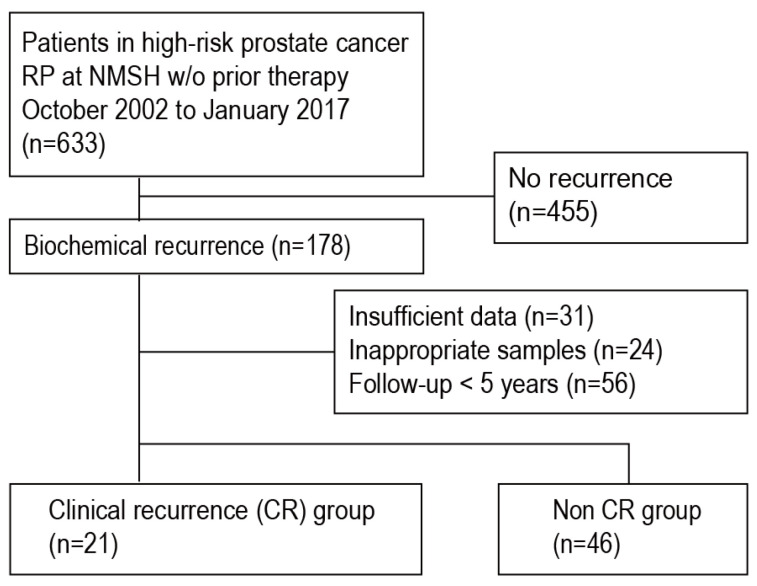
Flowchart of PC patient selection. Abbreviations: CR, clinical recurrence; NMSH, Nippon Medical School Hospital; RP, radical prostatectomy.

Between October 2002 and January 2017, 633 patients were diagnosed with HRPC and underwent open/laparoscopic/robot-assisted RP at Nippon Medical School Hospital (NMSH) without any prior therapy. Follow-up was scheduled at least every 3 months after surgery. An increase of at least 0.2 ng/mL in the PSA value was considered to indicate BCR. All such patients received salvage radiotherapy or hormonal adjuvant therapy at the clinician’s discretion. After RP, 178 patients experienced BCR (i.e., post-RP BCR in patients with HRPC). Of these, the numbers of patients who did and did not progress to CR were 24 and 154, respectively; CR was defined as metastatic disease confirmation on imaging studies (e.g., positron emission tomography/computed tomography and bone scintigraphy). Ineligible patients (for whom clinical or pathological information was inadequate, who underwent less than 5 years of follow-up after BCR, or whose tissue samples were inadequately stored) were excluded from the study. Finally, we enrolled 21 patients with CR (the CR group) and 46 non-CR patients with more than 5 years of follow-up after BCR (the non-CR group) when exploring candidate biomarkers for prediction of CR in patients with HRPC who experienced post-RP BCR. The patient selection criteria are presented in Figure 1, and the clinical characteristics of all patients are listed in Table 1. This study adhered to the 2013 Declaration of Helsinki and the principles of the Japanese Society of Pathology Ethics Committee. The NMSH Institutional Review Board approved this study (approval no. A-2020-049), and written informed consent was obtained from all patients.

### 2.2. Formalin-Fixed Paraffin-Embedded (FFPE) Radical Prostatectomy (RP) Specimens

A total of 67 FFPE RP specimens were obtained from the abovementioned 21 cases with CR and the 46 cases without CR in patients with HRPC who experienced post-RP BCR. RP samples were fixed in 20% formalin, sliced into approximately 3–5 mm thick slices perpendicular to the rectal surface from the apex of the prostate to the bladder neck side, and embedded in paraffin. Multiple hematoxylin-and-eosin-stained slides from these FFPE RP specimens were examined by pathologists of the Department of Clinical Pathology of NMSH to diagnose PC in accordance with the International Society of Urological Pathology (ISUP) grading system [13]. All FFPE samples were stored at room temperature for between 5 and 20 years before RNA isolation was performed. For RNA extraction, sections with a thickness of 10 μm and, thus, volumes of approximately 10 mm^3^ (e.g., four sections, each with an area of 250 mm^2^) were collected from the PC regions of the FFPE blocks using a microtome (catalog no. TU213; Yamato Kohki Industrial Co., Ltd., Saitama, Japan). The clinicopathological data of the 67 prostate cancer cases included in this study are listed in Supplementary Table S1. 

### 2.3. RNA Extraction

Total RNA was extracted using a RNeasy FFPE kit (catalog no. 73504; Qiagen, Hilden, Germany), in accordance with the manufacturer’s instructions. RNA quality was assessed using an Agilent RNA 6000 nano Kit (catalog no. 5067-1511; Agilent Technologies, Palo Alto, CA, USA) and a 2100 Bioanalyzer (Agilent Technologies). DNA was removed via DNase I treatment (2700 kU/mL, catalog no. 73504; Qiagen). RNA concentrations were determined with a Quantus fluorometer (catalog no. E6150; Promega Corporation, Madison, WI, USA). Ribosomal RNA (rRNA) was depleted from total RNA using an NEBNext rRNA depletion Kit v2 (catalog no. E7405; New England Biolabs, Ipswich, MA, USA).

### 2.4. RNA-sequencing (RNA-seq)

First, we performed a preliminary RNA-seq analysis to comprehensively profile RNA expression, using three FFPE samples from the CR group that matched the relevant criteria (a DV200 value of at least 30% of total extracted RNA, a single peak at approximately 300 bp in the bioanalyzer electrophoretic diagram, and a total RNA library concentration of at least 4 nM). After rRNA depletion, cDNA libraries were constructed using the NEBNext Ultra II Directional RNA Library Prep Kit for Illumina (catalog no. E7760; New England Biolabs), in accordance with the manufacturer’s protocol. All libraries were purified via the addition of AMPure XP magnetic beads (catalog no. A63811; Beckman Coulter, Pasadena, CA, USA); their qualities were assessed using an Agilent High Sensitivity DNA Kit (catalog no. 5067-4626; Agilent Technologies) and a 2100 Bioanalyzer. Sequencing was performed on an Illumina HiSeq X Ten platform (Illumina, San Diego, CA, USA) running a 150-cycle single-read protocol with a depth of approximately 200 million reads per sample. Read qualities were determined using the FastQC program (version 0.11.7; https://www.bioinformatics.babraham.ac.uk/projects/fastqc/ (accessed on 4 February 2022)); subsequent filtering and trimming were performed with Trimmomatic (version 0.38; http://www.usadellab.org/cms/?page=trimmomatic (accessed on 4 February 2022)). Reads were then mapped to the human genome version GRCh38.19 (NCBI_109.20200522) using HISAT2 software (version 2.1.0; http://daehwankimlab.github.io/hisat2/ (accessed on 4 February 2022)). FeatureCounts software (version 1.6.3; http://subread.sourceforge.net or http://www.bioconductor.org (accessed on 4 February 2022)) was used to count the numbers of reads that mapped to particular genes.

### 2.5. Quantitative Polymerase Chain Reaction (qPCR) Analysis

A qPCR amplifying RNAs was performed using a 7300 Real-Time PCR System (Applied Biosystems, Foster City, CA, USA) or a 7900 FAST Real-Time PCR System (Applied Biosystems). Briefly, total RNA was reverse transcribed with a PrimeScript RT reagent kit (catalog no. RR037A; TaKaRa Bio, Shiga, Japan). To quantify RNA expression levels, the reverse-transcription products were subjected to qPCR using TB Green Premix Ex Taq (catalog no. RR420A; TaKaRa Bio). The sequences of the *RNU1-1* and *RNU1-2* genes are identical, although the gene loci lie in different positions on chromosome 1. Thus, the two genes were regarded as a single gene (designated as RNU1-1/1-2), and an appropriate primer was constructed. To normalize the expression levels of RNAs (RNU1-1/1-2, *RNU4-1*, *RNU4-2*, and *PCA3*), *RN7SK-201* served as an endogenous internal control. *RN7SK-201* was consistently highly expressed across all FFPE samples of the aforementioned RNA-seq analysis (Supplementary Table S2 (transcript analysis)). The following primers (all 5′ to 3′) were used: RNU1-1/1-2 forward, GATCACGAAGGTGGTTTTCC, and reverse, CAGTCCCCCACTACCACAAA; *RNU4-1*: forward, CTATCCGAGGCGCGATTATT, and reverse, AAAATTGCCAGTGCCGACTA; *RNU4-2*: forward, TATCCGAGGCGCGATTATTG, and reverse, GTCAAAAATTGCCAATGCCGA; *PCA3*: forward, CAGAGGGGAGATTTGTGTGG, and reverse, CGTTTCAGTAGTGCCCCAGT; *RN7SK-201*: forward, CGGTCTTCGGTCAAGGGTAT, and reverse, CCCTACGTTCTCCTACAAATGG.

### 2.6. Statistical Analyses

All statistical analyses were performed with JMP software (version 13.2.0; SAS Institute, Cary, NC, USA). The characteristics of the two groups were compared using the Wilcoxon rank-sum test for continuous variables. Clinical recurrence-free survival (RFS) curves were generated by the Kaplan–Meier method and compared using the log-rank test. A multivariate Cox proportional hazards model was constructed with clinical RFS as the outcome variable; hazard ratios (for the CR group compared with the non-CR group), 95% confidence intervals, and *p*-values were calculated. qPCR data are expressed as means ± standard errors (SEs). A *p*-value < 0.05 was considered statistically significant.

## 3. Results

### 3.1. RNA-seq of FFPE RP Samples from Patients with HRPC Who Developed CR after Post-RP BCR

First, we conducted an RNA-seq analysis of three FFPE RP samples from the CR group to determine which RNAs were stable and detectable in FFPE samples. It has been suggested that RNAs from clinical FFPE samples exhibit poor quality (e.g., they are degraded) [34,35]. The clinicopathological characteristics of the three FFPE samples (nos. 1, 2, and 10) are listed in Supplementary Table S1. We obtained 6.98 million mapped reads, with a mean of 2.33 million mapped reads per sample. A total of 57,116 genes were detected via RNA-seq analysis (Supplementary Table S2 (gene analysis)); a summary is presented in Table 2. Protein-encoding RNA genes were the most abundant gene type detected via RNA-seq (i.e., 50.4% of all detected genes). In terms of non-coding RNA (ncRNA) genes, the relative abundances (in transcripts per million) of long ncRNA (lncRNA) genes and small ncRNA (i.e., miRNA, snRNA, and snoRNA) genes were 22.3% and 9.2% of all detected genes, respectively. The top 50 most highly expressed genes in FFPE samples are listed in Table 3. There were many snRNA and mitochondrial genes among the top 50 most highly expressed genes (Table 3). Genes of the nucleus and mitochondrion, as well as protein-encoding RNA genes in the cytosol, were well-preserved in FFPE samples [36,37]. 

### 3.2. Comparison of the Expression Levels of snRNAs between CR and Non-CR Groups Using qPCR

Intriguingly, many snRNA genes were included in the top 50 most highly expressed genes (Table 3), although snRNA genes constituted only 4.9% of all detected genes (in transcripts per million; Table 2). snRNAs primarily function to process pre-mRNAs in the nucleus [28,29,30], but their dysregulation has recently been reported in some cancers, indicating the potential importance of snRNAs as cancer biomarkers and therapeutic targets [31,32,33]. Therefore, we investigated whether snRNAs in FFPE RP samples were prognostic factors for CR in patients with HRPC who experienced post-RP BCR. We focused on the three most highly expressed snRNA genes (i.e., RNU1-1/1-2, *RNU4-1*, and *RNU4-2*; Supplementary Table S3) and compared their expression levels between CR (*n* = 21) and non-CR (*n* = 46) groups via qPCR of the FFPE RP samples (Table 1 and Supplementary Table S1). As mentioned above, because the *RNU1-1* and *RNU1-2* genes share the same sequence, the two transcripts were regarded as a single gene (designated as RNU1-1/1-2).

**Figure 2:**
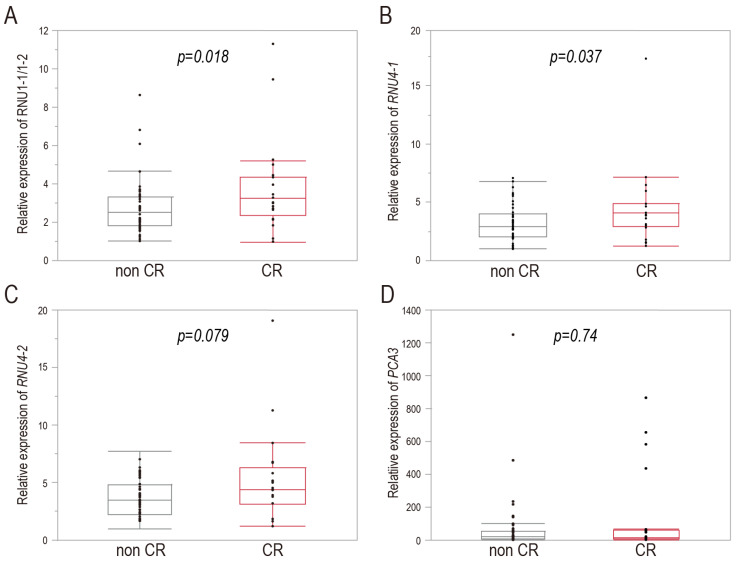
snRNA RNU1-1/1-2 and *RNU4-1* are upregulated in FFPE RP samples from patients with HRPC who develop CR after post-RP BCR. qPCR analyses of the RNU1-1/1-2 (**A**), *RNU4-1* (**B**), *RNU4-2* (**C**), and *PCA3* (**D**) levels in CR and non-CR groups. In the boxplots, the center lines are medians, box limits are 25th and 75th percentiles, and whiskers are 1.5 × the interquartile ranges from the 25th and 75th percentiles. *RN7SK-201* served as the internal control. Abbreviations: BCR, biochemical recurrence; CR, clinical recurrence; FFPE, formalin-fixed paraffin-embedded; HRPC, high-risk prostate cancer; RP, radical prostatectomy; snRNA, small nuclear RNA.

The qPCR revealed a significantly higher RNU1-1/1-2 expression in the CR group than in the non-CR group (3.88 ± 0.54 vs. 2.76 ± 0.22; *p* = 0.018; Figure 2A). *RNU4-1* expression was also significantly higher in the CR group than in the non-CR group (4.49 ± 0.73 vs. 3.19 ± 0.23; *p* = 0.037; Figure 2B). Conversely, there was no significant between-group difference in *RNU4-2* expression (5.31 ± 0.86 vs. 3.68 ± 0.26; *p* = 0.0879; Figure 2C). Additionally, we compared the expression levels of lncRNA *PCA3* between the two groups. *PCA3,* which is significantly overexpressed in PC patients, is one of the best-known biomarkers of PC [38,39]. Urine-based detection (i.e., the PCA3 test) is a helpful non-invasive method for PC diagnosis [40]. In our RNA-seq analysis, lncRNA *PCA3* was not an abundant gene (i.e., rating 516 of all 57,116 genes). There was no significant difference in terms of *PCA3* expression between the two groups (138.75 ± 56.11 vs. 74.65 ± 28.92; *p* = 0.74; Figure 2D). These qPCR results indicate that snRNA RNU1-1/1-2 and *RNU4-1* are candidate prognostic predictors of CR in patients with HRPC who experience post-RP BCR.

### 3.3. Correlations of RNU1-1/1-2 and RNU4-1 Expression with Clinicopathological Features of Patients with HRPC Who Experienced Post-RP BCR

Next, we sought correlations between RNU1-1/1-2 and *RNU4-1* expression and clinicopathological features of patients with HRPC who experienced post-RP BCR. The 67 samples were divided into two groups (i.e., high- and low-expression groups) according to the median values of the snRNA RNU1-1/1-2 and *RNU4-1* levels; these were 2.76 and 3.22, respectively. The clinicopathologically prognostic factors listed in Table 1 were regarded as dichotomous variables: age at RP (<70 years vs. ≥70 years), preoperative PSA level (<20 ng/mL vs. ≥20 ng/mL), ISUP Grade Group (3–4 vs. 5), pathological T stage (<3a vs. ≥3a), pathological N stage (negative vs. positive), and surgical margin status (negative vs. positive). No statistically significant correlations were observed between snRNA expression levels and these clinicopathological factors, with the exception of preoperative PSA level (Table 4).

### 3.4. Evaluation of the Prognostic Utilities of snRNA RNU1-1/1-2 and RNU4-1 for CR in Patients with HRPC Who Experienced Post-RP BCR

**Figure 3:**
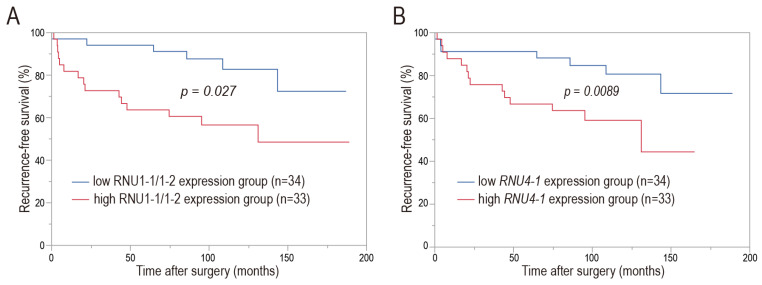
snRNA RNU1-1/1-2 and *RNU4-1* levels are significantly correlated with clinical recurrence-free survival (RFS) in patients with HRPC who experience post-RP BCR. Kaplan–Meier survival curves for clinical RFS according to levels of RNU1-1/1-2 (**A**) and *RNU4-1* (**B**) are presented. Abbreviations: BCR, biochemical recurrence; HRPC, high-risk prostate cancer RP, radical prostatectomy; snRNA, small nuclear RNA.

The relationship between snRNA expression levels (those of RNU1-1/1-2 and *RNU4-1*) and clinical recurrence-free survival (RFS) in patients with HRPC who experienced post-RP BCR was investigated; the median follow-up interval was 104 months (interquartile range (IQR), 22.1–66.8 months). The snRNA RNU1-1/1-2 and *RNU4-1* levels were significantly correlated with clinical RFS status in patients with HRPC who experienced post-RP BCR. During follow-up, 15 (45.4%) and 6 (17.6%) patients developed CR in the high- and low-RNU1-1/1-2 expression groups, respectively. Clinical RFS was significantly shorter in patients with high RNU1-1/1-2 levels than patients with low RNU1-1/1-2 levels (*p* = 0.0089, Figure 3A). With respect to the *RNU4-1* snRNA, 14 (42.4%) and 7 (20.6%) patients developed CR in the high- and low-*RNU4-1* groups, respectively. Patients with high-level *RNU4-1* expression exhibited significantly shorter clinical RFS compared with patients who had low-level expression (*p* = 0.027, Figure 3B).

Multivariate survival analysis was conducted to determine whether the two snRNAs (i.e., RNU1-1/1-2 and *RNU4-1*) were prognostic in terms of clinical RFS in patients with HRPC who experienced post-RP BCR. The dependent variables were CR and non-CR status; the independent variables were age at RP (≥70 years), preoperative PSA level (≥20 ng/mL), ISUP Grade Group (5), pathological T stage (≥3a), pathological N stage (positive), surgical margin status (positive), and the expression levels of the snRNAs. The hazard ratios, 95% confidence intervals (CIs), and *p*-values are summarized in Table 5. The RNU1-1/1-2 level was significantly prognostic for CR in patients with HRPC who experienced post-RP BCR (hazard ratio, 4.101; 95% CI, 1.177–16.587; *p* = 0.026), but the *RNU4-1* level and other covariates were not. The data thus revealed that snRNA RNU1-1/1-2 may serve as an independent prognostic factor for clinical RFS in patients with HRPC who experience post-RP BCR.

## 4. Discussion

BCR is common, such that approximately 26% of all PC patients experience BCR within 15 years after RP (the primary definitive treatment) [15]. BCR does not necessarily trigger CR; for patients with HRPC, BCR is associated with higher risks of distant metastasis and worse PC-specific mortality [15,17,41]. Therefore, in patients with HRPC who experience post-RP BCR, complementary prognostic biomarkers are required to identify the approximately 10% of all patients who develop CR during extended follow-up [15]. In this study, RNA-seq analysis demonstrated that many snRNA genes were very abundant in FFPE RP specimens from patients with HRPC who developed CR after post-RP BCR. The subsequent qPCR analysis of FFPE RP samples from patients with HRPC with and without CR after post-RP BCR revealed that the expression levels of snRNA RNU1-1/1-2 and *RNU4-1* (designated as prostate cancer-associated snRNAs) were significantly higher in patients with CR than in patients without CR (Figure 2). PC-associated snRNA levels were significantly correlated with clinical RFS in patients with HRPC who experienced post-RP BCR; patients exhibiting high-level expression of the snRNAs experienced significantly shorter clinical RFS compared with patients exhibiting low-level expression (Figure 3). Correlations between snRNA levels and several clinicopathological factors (e.g., preoperative PSA level, ISUP Grade Group, and tumor stage) were also investigated (Table 4); the absence of correlations between the snRNAs and these factors, with the exception of preoperative PSA level, suggest that the PC-associated snRNAs could provide unique prognostic information. Furthermore, the multivariate survival analysis showed that snRNA RNU1-1/1-2 might serve as an independent prognostic factor for clinical RFS in patients with HRPC who experienced post-RP BCR (Table 5). The utility of RNU1-1/1-2 as a biomarker is reinforced by its independent nature, especially in cases where known prognostic factors cannot distinguish between CR and non-CR patients.

Until recently, only a few studies had analyzed cancer-associated snRNAs (e.g., U2 snRNA fragments [RNU2-1f]) [42,43,44,45]. However, recent evidence indicates that aberrant snRNA expression induces tumorigenesis and cancer progression; snRNAs may serve as biomarkers of cancer prognosis and facilitate assessment of the treatment response [46,47,48,49]. Recent studies of cancer-associated snRNAs have highlighted the significance of U1 snRNA (*RNU1-1*). Highly recurrent hotspot mutations (U1 r.3A>G mutations) of U1 snRNA were primarily associated with sonic hedgehog medulloblastoma [48]. The U1 r.3A>G mutations drove 5′ cryptic alternative splicing, leading to inactivation of certain tumor-suppressor genes (e.g., *PTCH1*) [48]. Moreover, a highly recurrent A>C somatic mutation (i.e., g.3A>C) in U1 has been observed in patients with chronic lymphocytic leukemia (CLL) and hepatocellular carcinoma (HCC) [47]. This mutation created novel splice junctions and altered the splicing patterns of multiple genes, including known drivers of cancer (e.g., *MSI2*). The U1 g.3A>C mutation was associated with poor prognosis in patients exhibiting a more aggressive subtype of CLL. In addition to U1, N6-methyladenosine (m6A)-modified snRNAs (e.g., *RNU6-2*) were upregulated in HCC tissues compared with non-HCC tissues [31]. Prognostic risk scores in patients with HCC, established using the Cancer Genome Atlas (TCGA) database based on the m6A-associated snRNA model, independently predicted overall survival in HCC patients. snRNAs (e.g., *RNU6-1143P*) were also associated with the overall survival of acute myeloid leukemia patients in the TCGA cohort [32]. Low-level expression of *RNU5E-1*, a novel variant of U5 snRNA, was independently associated with improved tumor-free survival and long-term survival in patients with HCC [33]. In the present study, we showed that aberrant expressions of snRNA RNU1-1/1-2 and *RNU4-1* could serve as potential indicators of the prognosis in patients with HRPC who experience post-RP BCR. To our knowledge, this is the first report of PC-associated snRNAs.

The lncRNA *PCA3* (specific to the prostate) is significantly overexpressed in PC [38,39], and its urine-based detection (i.e., the PCA3 test) is a valuable non-invasive method for PC diagnosis [40]. However, the relationships of PCA3 status, aggressive features of PC, and treatment outcomes remain unclear; the evidence is conflicting [39]. Merola et al. reported that higher urine PCA3 scores were associated with greater tumor aggressiveness (GS ≥ 7) [50]. Conversely, Alshalalfa et al. reported that low-level PCA3 expression was associated with high Gleason grades (4 and 5) of biopsy and RP tissues; it was also correlated with a higher risk of metastasis and more aggressive PC after RP [51]. We found no significant difference in *PCA3* expression between FFPE RP tissues of the CR and non-CR groups (Figure 2D). Thus, the lncRNA *PCA3* is unlikely to be involved in PC clinical recurrence after PR. In terms of the RNAs detected via RNA-seq of FFPE RP samples, several snRNA and snoRNA genes were among the top 50 most highly expressed genes (Table 3); however, the snRNA and snoRNA genes constituted only 4.9% and 2.0% of all genes (in transcripts per million), respectively (Table 2). snoRNAs, as well as snRNAs, are involved in tumorigenesis and cancer progression; snoRNAs have potential as useful diagnostic biomarkers and therapeutic targets in patients with various cancers [52,53,54]. Further work is needed to determine whether aberrantly expressed snoRNAs are correlated with PC progression after RP.

Our work had some limitations. First, the sample size, especially for the CR group, was limited, and the research was conducted at a single institution. A multicenter cohort study is needed to validate our results. Second, we used FFPE samples. FFPE tissue processing and storage can trigger RNA degradation, fragmentation, and modification, all of which may affect the quality and reliability of RNA-seq and qPCR data [35,36,55]. Thus, we used FFPE RNA extraction and library preparation methods that were specifically developed to improve the reliability of RNA-seq and qPCR data from FFPE-derived RNA samples [56,57]. Although the results obtained from FFPE samples should be interpreted with caution, small ncRNAs (e.g., snRNAs and snoRNAs) are less likely to be adversely affected by FFPE sample preparation and storage compared with coding RNAs and lncRNAs [58]. Additionally, the ways in which the PC-associated snRNAs identified in this study affect the molecular mechanisms of PC progression after RP require further investigation.

## 5. Conclusions

In conclusion, our RNA-seq and qPCR analyses, using FFPE RP specimens, yielded important information concerning novel, potentially prognostic factors for CR in patients with HRPC who experience post-RP BCR. In this study, we discovered that snRNA RNU1-1/1-2 was significantly upregulated in FFPE RP samples from patients with HRPC who developed CR after post-RP BCR. In such patients, there was a significant correlation between the snRNA RNU1-1/1-2 level and clinical RFS. snRNA RNU1-1/1-2 could serve as an independent prognostic factor for clinical RFS in patients with HRPC who experience post-RP BCR. Our findings offer new insights into the involvement of snRNAs in PC progression.

## Figures and Tables

**Table 1:** Clinical characteristics of patients with high-risk prostate cancer (HRPC) with and without clinical recurrence (CR) after post-radical prostatectomy biochemical recurrence (post-RP BCR).

Feature		CR (*n* = 21)	Non-CR (Control) (*n* = 46)	Total (*n* = 67)	*p*-Value
Age at RP, no. (%)					0.36
	<70	16 (76.2%)	30 (65.2%)	46 (68.7%)	
	≥70	5 (23.8%)	16 (34.8%)	21 (31.3%)	
Preoperative PSA, no. (%)					0.80
	<20	13 (61.9%)	27 (58.7%)	40 (59.7%)	
	≥20	8 (38.1%)	19 (41.3%)	27 (40.3%)	
ISUP Grade Groups, no. (%)					0.24
	ISUP 3-4	9 (42.9%)	13 (28.2%)	22 (32.8%)	
	ISUP 5	12 (57.1%)	33 (71.7%)	45 (67.2%)	
pT, no. (%)					0.36
	<3a	1 (4.8%)	7 (15.2%)	7 (15.2%)	
	≥3a	20 (95.2%)	39 (84.8%)	39 (84.8%)	
pN, no. (%)					0.58
	negative	20 (95.2%)	45 (97.8%)	45 (97.8%)	
	positive	1 (4.8%)	1 (2.2%)	1 (2.2%)	
Surgical margin, no. (%)					0.65
	negative	4 (19.0%)	11 (23.9%)	11 (23.9%)	
	positive	17 (81.0%)	35 (76.1%)	35 (76.1%)	

Abbreviations: CR, clinical recurrence; ISUP, International Society of Urological Pathology; PSA, prostate specific antigen; RP, radical prostatectomy; pN, pathological N stage; pT, pathological T stage.

**Table 2:** Genes detected by RNA-seq of FFPE RP samples from patients with HRPC who developed CR after post-RP BCR (gene analysis).

Gene Type	The Number of Genes, No. (%)	TPM, No. (%) ^†^
Protein coding	19,460 (34.1%)	503,797.7 (50.4%)
lncRNA	17,323 (30.3%)	223,407.3 (22.3%)
Processed pseudogene	9503 (16.6%)	72,746.4 (7.3%)
Unprocessed pseudogene	2303 (4.0%)	13,639.7 (1.4%)
miscRNA	1788 (3.1%)	52,583.2 (5.3%)
snRNA	1436 (2.5%)	48,532.3 (4.9%)
miRNA	1077 (1.9%)	23,432.7 (2.3%)
snoRNA	586 (1.0%)	19,780.2 (2.0%)
IG gene	178 (0.3%)	1858.7 (0.2%)
IG pseudogene	161 (0.3%)	690.1 (0.07%)
rRNA	25 (0.04%)	343.2 (0.03%)
Others	3276 (5.7%)	39,188.3 (3.9%)
Total	57,116	1,000,000

† Transcripts per million (TPM), representing the relative abundance of a transcript among a population of sequenced transcripts. Abbreviations: BCR, biochemical recurrence; CR, clinical recurrence; FFPE, formalin-fixed paraffin-embedded; HRPC, high-risk prostate cancer; IG, immunoglobulin; lncRNA, long non cording RNA; miRNA, microRNA; miscRNA, miscellaneous RNA; RP, radical prostatectomy; rRNA, ribosomal RNA; snRNA, small nuclear RNA; snoRNA, small nucleolar RNA.

**Table 3:** The top 50 most highly expressed genes detected by RNA-seq of FFPE RP samples from patients with HRPC who developed CR after post-RP BCR (gene analysis).

No.	Gene ID	Gene Symbol	Gene Type	TPM, No. (%) ^†^
1	ENSG00000251562	*MALAT1*	lncRNA	35,265.9 (3.5%)
2	ENSG00000276168	*RN7SL1*	miscRNA	9968.7 (1.0%)
3	ENSG00000202538	*RNU4-2*	snRNA	9476.2 (0.9%)
4	ENSG00000142515	*KLK3*	protein coding	6448.2 (0.6%)
5	ENSG00000198695	*MT-ND6*	protein coding	6274.2 (0.6%)
6	ENSG00000200488	*RN7SKP203*	miscRNA	6039.1 (0.6%)
7	ENSG00000198886	*MT-ND4*	protein coding	5699.7 (0.6%)
8	ENSG00000198727	*MT-CYB*	protein coding	4566.4 (0.5%)
9	ENSG00000198899	*MT-ATP6*	protein coding	4407.9 (0.4%)
10	ENSG00000198938	*MT-CO3*	protein coding	4093.9 (0.4%)
11	ENSG00000198804	*MT-CO1*	protein coding	4015.8 (0.4%)
12	ENSG00000245532	*NEAT1*	lncRNA	3728.9 (0.4%)
13	ENSG00000206652	*RNU1-1*	snRNA	3678.7 (0.4%)
14	ENSG00000200087	*SNORA73B*	snoRNA	3582.9 (0.4%)
15	ENSG00000198786	*MT-ND5*	protein coding	3456.7 (0.3%)
16	ENSG00000167751	*KLK2*	protein coding	3348.0 (0.3%)
17	ENSG00000198840	*MT-ND3*	protein coding	3297.2 (0.3%)
18	ENSG00000198712	*MT-CO2*	protein coding	2767.5 (0.3%)
19	ENSG00000198888	*MT-ND1*	protein coding	2565.9 (0.3%)
20	ENSG00000198763	*MT-ND2*	protein coding	2430.4 (0.2%)
21	ENSG00000278771	*RN7SL3*	miscRNA	2237.6 (0.2%)
22	ENSG00000201098	*RNY1*	miscRNA	2057.4 (0.2%)
23	ENSG00000200795	*RNU4-1*	snRNA	1954.3 (0.2%)
24	ENSG00000212907	*MT-ND4L*	protein coding	1727.5 (0.2%)
25	ENSG00000228253	*MT-ATP8*	protein coding	1707.9 (0.2%)
26	ENSG00000238741	*SCARNA7*	snoRNA	1650.4 (0.2%)
27	ENSG00000265735	*RN7SL5P*	miscRNA	1612.6 (0.2%)
28	ENSG00000273149	antisense to *TPT1*	lncRNA	1602.5 (0.2%)
29	ENSG00000207005	*RNU1-2*	snRNA	1523.2 (0.2%)
30	ENSG00000277918	*RNVU1-28*	snRNA	1519.4 (0.2%)
31	ENSG00000204389	*HSPA1A*	protein coding	1454.1 (0.1%)
32	ENSG00000110092	*CCND1*	protein coding	1453.5 (0.1%)
33	ENSG00000272114	antisense to *VEGFA*	lncRNA	1400.1 (0.1%)
34	ENSG00000158715	*SLC45A3*	protein coding	1308.6 (0.1%)
35	ENSG00000204388	*HSPA1B*	protein coding	1283.2 (0.1%)
36	ENSG00000221792	*MIR1282*	miRNA	1277.8 (0.1%)
37	ENSG00000267458	antisense to *CALR*	lncRNA	1238.2 (0.1%)
38	ENSG00000266019	*MIR3609*	miRNA	1232.5 (0.1%)
39	ENSG00000200156	*RNU5B-1*	snRNA	1122.4 (0.1%)
40	ENSG00000263740	*RN7SL4P*	miscRNA	1083.8 (0.1%)
41	ENSG00000248527	*MTATP6P1*	unprocessed pseudogene	1022.2 (0.1%)
42	ENSG00000080824	*HSP90AA1*	protein coding	1017.9 (0.1%)
43	ENSG00000207389	*RNU1-4*	snRNA	983.5 (0.1%)
44	ENSG00000202058	*RN7SKP80*	miscRNA	951.9 (0.1%)
45	ENSG00000256364	antisense to *MLEC*	lncRNA	925.9 (0.1%)
46	ENSG00000112306	*RPS12*	protein coding	909.4 (0.1%)
47	ENSG00000286037	antisense to *SPINT2*	lncRNA	903.2 (0.1%)
48	ENSG00000200312	*RN7SKP255*	miscRNA	878.9 (0.1%)
49	ENSG00000167034	*NKX3-1*	protein coding	873.3 (0.1%)
50	ENSG00000096384	*HSP90AB1*	protein coding	856.7 (0.1%)

^†^ Transcripts per million (TPM), representing the relative abundance of a transcript among a population of sequenced transcripts. Abbreviations: BCR, biochemical recurrence; CR, clinical recurrence; FFPE, formalin-fixed paraffin-embedded; HRPC, high-risk prostate cancer; IG, immunoglobulin; lncRNA, long non cording RNA; miRNA, microRNA; miscRNA, miscellaneous RNA; RP, radical prostatectomy; rRNA, ribosomal RNA; snRNA, small nuclear RNA; snoRNA, small nucleolar RNA.

**Table 4:** The correlation of snRNA RNU1-1/1-2 and *RNU4-1* expression with clinicopathological features of patients with HRPC who experienced post-RP BCR.

Variable	Group	RNU1-1/1-2 Expression		*p*-Value	*RNU4-1* Expression		*p*-Value
		Low	High		Low	High	
Age at RP, no. (%)				0.48			0.08
	<70 y/o	22 (64.7%)	24 (72.7%)		20 (58.8%)	26 (78.8%)	
	≥70 y/o	12 (35.3%)	9 (27.3%)		14 (41.2%)	7 (21.2%)	
Preoperative PSA, no. (%)				0.02			0.40
	<20 ng/mL	25 (73.5%)	15 (45.5%)		22 (64.7%)	18 (54.5%)	
	≥20 mg/mL	9 (26.5%)	18 (54.5%)		12 (35.3%)	15 (45.5%)	
ISUP Grade Groups, no. (%)				0.34			0.34
	3–4	13 (38.2%)	9 (27.3%)		13 (38.2%)	9 (27.3%)	
	5	21 (61.8%)	24 (72.7%)		21 (61.8%)	24 (72.7%)	
pT, no. (%)				0.48			0.13
	<3a	5 (14.7%)	3 (9.1%)		6 (17.7%)	2 (6.1%)	
	≥3a	29 (85.3%)	30 (90.1%)		28 (82.4%)	31 (93.9%)	
pN, no. (%)				0.98			0.09
	negative	33 (97.1%)	32 (97.0%)		34 (100%)	31 (93.9%)	
	positive	1 (2.9%)	1 (3.0%)		0 (0%)	2 (6.1%)	
Surgical margin, no. (%)				0.41			0.41
	negative	9 (26.5%)	6 (18.2%)		9 (26.5%)	6 (18.2%)	
	positive	25 (73.5%)	27 (81.8%)		25 (73.5%)	27 (81.8%)	

Abbreviations: BCR, biochemical recurrence; HRPC, high-risk prostate cancer ISUP, International Society of Urological Pathology; PSA, prostate specific antigen; RP, radical prostatectomy; pN, pathological N stage; pT, pathological T stage; snRNA, small nuclear RNA.

**Table 5:** Multivariate Cox regression analysis of prognostic factors contributing to clinical RFS in patients with HRPC who experienced post-RP BCR.

Covariates	Hazard Ratio	95% CI	*p*-Value
Age ≥ 70	0.587	0.164–1.683	0.34
Preoperative PSA ≥ 20 ng/mL	0.647	0.219–1.755	0.40
ISUP Grade Group 5	0.701	0.252–1.943	0.49
pT ≥ 3a	2.709	0.522–49.839	0.28
pN positive	8.806	0.374–91.963	0.15
Surgical margin positive	1.070	0.322–4.199	0.92
RNU1-1/1-2 expression level	4.101	1.177–16.587	0.03
*RNU4-1* expression level	0.972	0.282–3.460	0.96

Abbreviations: BCR, biochemical recurrence; CI, confidence interval; HRPC, high-risk prostate cancer; ISUP, International Society of Urological Pathology; PSA, prostate-specific antigen; RP, radical prostatectomy; pN, pathological N stage; pT, pathological T stage.

## Data Availability

All data generated or analyzed during this study are included in this published article and its Supplementary Materials.

## Supplementary Materials

The following supporting information can be downloaded at https://www.mdpi.com/article/10.3390/cancers16091757/s1, Supplementary Table S1: Clinicopathological characteristics of 67 patients with HRPC who experienced BCR after RP; Supplementary Table S2: Genes and transcripts detected by RNA-seq of the FFPE RP samples from patients with HRPC who developed CR after post-RP BCR; Supplementary Table S3: The top 50 most highly expressed snRNA genes detected by RNA-seq of the FFPE RP samples from patients with HRPC who developed CR after post-RP BCR.
