# Supplementary material for: snRNAs from Radical Prostatectomy Specimens Have the Potential to Serve as Prognostic Factors for Clinical Recurrence after Biochemical Recurrence in Patients with High-Risk Prostate Cancer
Source: Cancers (Basel). 2024 May 1;16(9):1757. doi: 10.3390/cancers16091757 (PMC11083327; doi:10.3390/cancers16091757)
Supplement: Supplementary file 1 [file cancers-16-01757-s001.zip › Suppl Table S1 R1 ver1.pdf]

Supplementary Table S1. Clinicopathological characteristics of 67 patients with HRPC who experienced BCR after RP (post-RP BCR).

No.	Group	Age at RP	PSA (ng/ml)	ISUP grading group	pT	pN	RM	Metastasis	Time to BCR from RP (month)	Time to CR from RP (month)
1	CR	69	18.2	5	3b	0	1	OSS	0.0	0.0
2	CR	72	99.0	5	3b	1	1	PUL, LYM	0.0	1.4
3	CR	69	12.0	5	3a	0	1	OSS	0.0	42.8
4	CR	73	28.1	5	4	0	1	PUL, LYM. OSS	0.0	47.9
5	CR	70	15.0	5	4	0	1	OSS	5.2	64.7
6	CR	67	17.0	4	2b	0	0	OSS	32.1	143.5
7	CR	58	16.0	5	3b	0	1	LYM	10.2	20.4
8	CR	63	7.3	5	3b	0	0	LYM	12.5	21.3
9	CR	59	37.3	5	3a	0	1	OSS	0.0	4.4
10	CR	66	14.5	5	3a	0	1	LYM OSS	0.0	3.6
11	CR	58	37.2	5	3a	0	0	OSS	12.4	44.4
12	CR	64	16.8	3	3a	0	0	LYM, OSS	0.0	22.4
13	CR	60	5.9	4	4	0	1	LYM, OSS	0.0	5.1
14	CR	70	52.4	3	4	0	1	LYM	14.1	16.8
15	CR	61	32.9	5	4	0	1	OSS	0.0	7.8
16	CR	53	28.7	4	4	0	1	LYM, OSS	0.0	85.8
17	CR	59	20.8	4	4	0	1	OSS	0.0	3.9
18	CR	81	13.7	5	3b	0	1	LYM, OSS	34.8	74.7
19	CR	68	15.0	4	3a	0	1	LYM	37.3	108.7
20	CR	68	7.1	4	3a	0	1	OSS	88.1	95.3
21	CR	69	9.3	4	3b	0	1	OSS	15.2	131.1
22	non-CR	63	11.3	5	3b	0	1		24.0	
23	non-CR	68	86.6	5	3b	0	1		4.0	
24	non-CR	63	165.1	5	3b	0	1		7.0	
25	non-CR	62	26.3	5	4	0	1		21.0	
26	non-CR	64	69.1	5	4	0	1		12.0	
27	non-CR	60	13.6	5	3a	0	0		28.0	
28	non-CR	75	33.5	5	4	0	1		3.0	
29	non-CR	71	16.4	5	3b	0	1		2.0	
30	non-CR	68	7.2	5	2c	0	1		15.0	
31	non-CR	75	8.7	5	3a	1	1		1.0	
32	non-CR	76	7.4	5	3a	0	0		25.0	
33	non-CR	64	62.4	4	3b	0	1		5.0	
34	non-CR	62	40.1	5	3a	0	0		2.0	
35	non-CR	68	9.2	5	2c	0	1		11.0	
36	non-CR	68	6.4	4	3a	0	1		6.0	
37	non-CR	64	67.4	5	2c	0	1		19.0	
38	non-CR	71	12.9	4	3b	0	1		40.0	
39	non-CR	64	29.0	5	3a	0	0		16.0	
40	non-CR	64	7.4	5	3b	0	0		5.0	
41	non-CR	64	15.67	5	3a	0	1		34.0	
42	non-CR	70	34.2	5	4	0	1		45.0	
43	non-CR	73	47.5	5	3b	0	0		9.0	
44	non-CR	59	77.32	5	3b	0	1		5.0	

45	non-CR	49	12.1	5	2b	0	0	20.0
46	non-CR	73	17.9	5	2b	0	0	24.0
47	non-CR	65	36.2	5	3b	0	1	10.0
48	non-CR	65	81.7	5	3b	0	1	1.0
49	non-CR	69	9.6	4	3b	0	1	60.0
50	non-CR	70	218.9	4	4	0	1	22.0
51	non-CR	61	15.9	4	4	0	1	0.0
52	non-CR	63	12.1	5	3a	0	1	12.0
53	non-CR	74	16.2	5	4	0	1	3.0
54	non-CR	64	94.3	4	4	0	1	16.0
55	non-CR	54	10.4	5	3a	0	0	37.0
56	non-CR	59	7.9	4	2b	0	1	21.0
57	non-CR	72	170	5	3a	0	1	46.0
58	non-CR	63	6.8	5	3a	0	1	42.0
59	non-CR	50	14.1	4	3b	0	1	36.0
60	non-CR	70	3.3	4	3b	0	1	58.0
61	non-CR	73	8.4	4	3a	0	1	6.0
62	non-CR	74	14.2	4	3a	0	1	5.0
63	non-CR	64	25.1	4	3a	0	1	1.0
64	non-CR	71	29.2	5	2b	0	1	0.0
65	non-CR	65	16.3	5	3b	0	0	5.0
66	non-CR	70	8.4	5	3a	0	1	7.0
67	non-CR	68	10.0	5	3a	0	0	46.0

Abbreviation: BCR, biochemical recurrence; CR, clinical recurrence; HRPC, high-risk prostate cancer; ISUP, International Society of Urological Pathology; LYM, lymph node metastasis; OSS, osseous metastasis; PSA, prostate specific antigen; pN, pathological T stage; pT, pathological T stage; PUL, pulmonary metastasis; RP, radical prostatectomy; RM, resection margin.
