# Supplementary material for: snRNAs from Radical Prostatectomy Specimens Have the Potential to Serve as Prognostic Factors for Clinical Recurrence after Biochemical Recurrence in Patients with High-Risk Prostate Cancer
Source: Cancers (Basel). 2024 May 1;16(9):1757. doi: 10.3390/cancers16091757 (PMC11083327; doi:10.3390/cancers16091757)
Supplement: Supplementary file 1 [file cancers-16-01757-s001.zip › Suppl Table S3 R1 ver3.pdf]

Supplementary Table S3. The top 50 most highly expressed snRNA genes detected by RNA-seq of the FFPE RP samples from patients with HRPC who developed CR after post-RP BCR (gene analysis).

No.	Gene ID	Gene symbol	TPM, no. (%) [†]
1	ENSG00000202538	*RNU4-2*	9,476.2 (0.95%)
2	ENSG00000206652	*RNU1-1*	3,678.7 (0.37%)
3	ENSG00000200795	*RNU4-1*	1,954.3 (0.20%)
4	ENSG00000207005	*RNU1-2*	1,523.2 (0.15%)
5	ENSG00000277918	*RNVU1-28*	1,519.4 (0.15%)
6	ENSG00000200156	*RNU5B-1*	1,122.4 (0.11%)
7	ENSG00000207389	*RNU1-4*	983.5 (0.10%)
8	ENSG00000206585	*RNVU1-7*	838.4 (0.08%)
9	ENSG00000206737	*RNVU1-18*	717.7 (0.07%)
10	ENSG00000270722	*RNVU1-31*	622.1 (0.06%)
11	ENSG00000199568	*RNU5A-1*	616.1 (0.06%)
12	ENSG00000238554	*RNU1-88P*	402.7 (0.04%)
13	ENSG00000252498	*RNU6-1016P*	291.1 (0.03%)
14	ENSG00000206762	*RNU6-418P*	258.8 (0.03%)
15	ENSG00000221676	*RNU6ATAC*	241.6 (0.02%)
16	ENSG00000264229	*RNU4ATAC*	236.7 (0.02%)
17	ENSG00000273768	*RNVU1-29*	224.7 (0.02%)
18	ENSG00000239119	*RNU7-119P*	220.5 (0.02%)
19	ENSG00000252645	*RNU7-111P*	218.1 (0.02%)
20	ENSG00000274210	*RNVU1-27*	210.1 (0.02%)
21	ENSG00000238829	*RNU7-45P*	150.6 (0.02%)
22	ENSG00000200665	*RNU6-1188P*	149.6 (0.01%)
23	ENSG00000207175	*RNU1-67P*	145.5 (0.01%)
24	ENSG00000253054	*RNU7-77P*	145.0 (0.01%)
25	ENSG00000200882	*RNU6-681P*	142.9 (0.01%)
26	ENSG00000251870	*RNU2-69P*	142.7 (0.01%)
27	ENSG00000201198	*RNU6-879P*	141.0 (0.01%)
28	ENSG00000277610	*RNVU1-4*	127.6 (0.01%)
29	ENSG00000239151	*RNU7-195P*	126.9 (0.01%)
30	ENSG00000251718	*RNU2-13P*	123.8 (0.01%)
31	ENSG00000252796	*RNU7-11P*	121.9 (0.01%)
32	ENSG00000200814	*RNU6-595P*	121.5 (0.01%)
33	ENSG00000238719	*RNU7-96P*	118.3 (0.01%)
34	ENSG00000278099	*RNVU1-2A*	116.9 (0.01%)
35	ENSG00000238386	*RNU7-48P*	116.2 (0.01%)
36	ENSG00000251712	*RNU7-20P*	115.6 (0.01%)
37	ENSG00000272215	*U7*	111.5 (0.01%)
38	ENSG00000283509	*U6*	106.6 (0.01%)
39	ENSG00000212327	*RNU6-882P*	103.9 (0.01%)
40	ENSG00000212457	*RNU6-644P*	96.9 (0.01%)
41	ENSG00000202017	*RNU6-806P*	95.9 (0.01%)
42	ENSG00000252452	*RNU6-107P*	94.9 (0.01%)
43	ENSG00000252431	*RNU6-1247P*	89.9 (0.01%)
44	ENSG00000201616	*RNU1-91P*	89.3 (0.01%)

45	ENSG00000212332	*RNU6-780P*	88.7 (0.01%)
46	ENSG00000252756	*RNU6-577P*	87.8 (0.01%)
47	ENSG00000199872	*RNU6-942P*	86.6 (0.01%)
48	ENSG00000222610	*RNU6-402P*	86.2 (0.01%)
49	ENSG00000272160	*RNU4-5P*	85.6 (0.01%)
50	ENSG00000199512	*RNU6-212P*	83.8 (0.01%)

†: Transcripts per million (TPM) represents the relative abundance of a transcript among a population of sequenced transcripts
Abbreviation: BCR, biochemical recurrence; CR, clinical recurrence; FFPE, formalin-fixed paraffin-embedded; HRPC, high-risk prostate cancer; RP, radical prostatectomy; snRNA, small nuclear RNA.
